# Bioinformatic gene analysis for potential therapeutic targets of Huntington’s disease in pre-symptomatic and symptomatic stage

**DOI:** 10.1186/s12967-020-02549-9

**Published:** 2020-10-14

**Authors:** Chunchen Xiang, Shengri Cong, Bin Liang, Shuyan Cong

**Affiliations:** 1 Department of Neurology, Shengjing Hospital of China Medical University, 36 Sanhao Street, Heping District, Shenyang, 110004 Liaoning People’s Republic of China; 2 Bioinformatics of Department, School of Life Sciences, China Medical University, Shenyang, China

**Keywords:** Bioinformatics analysis, Biomarkers, miRNAs, Pre-symptomatic HD

## Abstract

**Background:**

Huntington’s disease (HD) is a neurodegenerative disorder characterized by psychiatric symptoms, serious motor and cognitive deficits. Certain pathological changes can already be observed in pre-symptomatic HD (pre-HD) patients; however, the underlying molecular pathogenesis is still uncertain and no effective treatments are available until now. Here, we reanalyzed HD-related differentially expressed genes from the GEO database between symptomatic HD patients, pre-HD individuals, and healthy controls using bioinformatics analysis, hoping to get more insight in the pathogenesis of both pre-HD and HD, and shed a light in the potential therapeutic targets of the disease.

**Methods:**

Pre-HD and symptomatic HD differentially expressed genes (DEGs) were screened by bioinformatics analysis Gene Expression Omnibus (GEO) dataset GSE1751. A protein–protein interaction (PPI) network was used to select hub genes. Subsequently, Gene Ontology (GO) enrichment analysis of DEGs and Kyoto Encyclopedia of Genes and Genomes (KEGG) analysis of hub genes were applied. Dataset GSE24250 was downloaded to verify our hub genes by the Kaplan–Meier method using Graphpad Prism 5.0. Finally, target miRNAs of intersected hub genes involved in pre-HD and symptomatic HD were predicted.

**Results:**

A total of 37 and 985 DEGs were identified in pre-HD and symptomatic HD, respectively. The hub genes, SIRT1, SUZ12, and PSMC6, may be implicated in pre-HD, and the hub genes, FIS1, SIRT1, CCNH, SUZ12, and 10 others, may be implicated in symptomatic HD. The intersected hub genes, SIRT1 and SUZ12, and their predicted target miRNAs, in particular miR-22-3p and miR-19b, may be significantly associated with pre-HD.

**Conclusion:**

The PSMC6, SIRT1, and SUZ12 genes and their related ubiquitin-mediated proteolysis, transcriptional dysregulation, and histone metabolism are significantly associated with pre-HD. FIS1, CCNH, and their related mitochondrial disruption and transcriptional dysregulation processes are related to symptomatic HD, which might shed a light on the elucidation of potential therapeutic targets in HD.

## Background

Huntington’s disease (HD) is an inherited neurodegenerative disorder characterized by progressive motor and cognitive deficits [1]. The pathogenesis of HD is associated with the abnormal accumulation of mutant huntingtin (mHtt), a protein with expansion of CAG repeats at the amino-terminus of wild-type huntingtin (Htt) [2]. Currently, effective therapies are lacking for HD [3]. In addition, neuronal degeneration can be observed in pre-symptomatic patients as early as 20 years prior to diagnosis [4, 5], indicating that therapeutic intervention could occur several years before the manifestation of clinical symptoms.

Prediction results of gene expression profiling and bioinformatics analysis have become increasingly useful in the diagnosis and therapy of various diseases [6–8], including those of the nervous system [9]. In addition to being used to identify the functional connections among genes in an unbiased manner for the in-depth study of biological processes, it can also be used to predict up- and downstream genes and to explore the relationship between gene expression and disease phenotypes [10, 11].

MicroRNAs (miRNAs, 19–24 nucleotides in length) are the most abundant and representative small non-coding RNAs, which negatively regulate messenger RNA (mRNA) levels by binding to the 3′-untranslated region [12, 13]. Numerous studies have described dysregulation of miRNAs in neurodegenerative diseases, indicating that miRNAs may useful in many diseases, including HD [14, 15].

In the present study, the gene expression profile, GSE1751, from the GEO database was reanalyzed with respect to HD-related differentially expressed genes (DEGs) in whole blood samples between symptomatic HD patients, pre-symptomatic HD (pre-HD) individuals, and healthy controls using bioinformatics analysis. Hub genes were selected for deeper functional analysis by constructing a protein–protein interaction (PPI) network and used to predict their related miRNAs. As a result, sets of genes and miRNAs relating to the pathogenesis of HD were identified, increasing our understanding of the disease process and shedding light on markers for the treatment of HD.

## Methods

### Data acquisition

The gene expression profile, GSE1751, from the GEO database was downloaded (https://www.ncbi.nlm.nih.gov/geo, May 18, 2017). GSE1751 is based on the GPL96 platform (Affymetrix Human Genome U133 Plus 2.0 Array) and contains a total of 31 peripheral whole blood samples, including 12 symptomatic HD cases, 5 pre-symptomatic HD cases, and 14 healthy controls [16]. People who are carriers of the HD mutation but have no clinically present signs or symptoms are considered pre-symptomatic cases [3].

### Screening of differentially expressed genes (DEGs)

The R package, “limma” (https://cran.r-project.org/), obtained from https://www.bioconductor.org [17], was used to analyze the GSE1751 raw expression data. Background correction, quantile normalization, and log2-transformation were performed to create a robust multi-array average (RMA) and a log-transformed perfect match. The Benjamini–Hochberg method was used to adjust original *p*-values, and the false discovery rate (FDR) procedure was used to calculate fold changes (FC). The DEGs between symptomatic HD (GSM30530 to GSM30541) and healthy control (GSM30580 to GSM30593) groups and between pre-symptomatic HD (GSM30542 to GSM30546) and healthy control groups were evaluated. The differential gene expression threshold was log2 fold change > 2 and *p*-value < 0.05.

### Integration of the protein–protein interaction (PPI) network and screening of the hub genes

The Search Tool for the Retrieval of Interacting Gene (STRING) database [18] was used to construct the PPI network of the DEGs in the two groups. Subsequently, the Cytoscape [19] software was used to build a PPI network, employing the Molecular Complex Detection (MCODE) plug-in to screen PPI network modules for these two groups of DEGs. The cluster method (No. 14) was used to cluster the modules, and the genes with an MCODE score ≥ 2 were selected as hub genes.

### Gene Ontology and KEGG pathway analysis of DEGs

Database for Annotation, Visualization, and Integrated Discovery (DAVID) (https://david.abcc.ncifcrf.gov/) [20] is a gene functional annotation tool that is helpful for understanding biological functions. GO (Gene Ontology) function enrichment analysis of the DEGs in the two groups was performed, and those with a *p*-value < 0.05 were considered significantly enriched. Subsequently, the Kyoto Encyclopedia of Genes and Genomes (KEGG) database was employed to elucidate the KEGG pathways of the hub genes in the two groups.

### Prediction of the miRNAs of the intersected hub genes and their validation

Finally, these hub genes were input into Targetscan [21] (https://www.targetscan.org/vert_71/) and miRDB [22] (https://www.mirdb.org/) to predict their possible miRNAs. The intersection portion of the two databases was selected as our predicted miRNAs. Moreover, based on the information in the individual MCODE modules, the node with the highest score was selected as the hub gene in GSE1751. Each hub gene was also found in the independent datasets (dataset GSE24250, HD samples n = 8, and HC samples n = 6) based on the downloaded raw data files, including the gene expression level, survival time, and survival state. Expression levels were divided into two groups, the HD group and the control group, according to X-tile [23]. The Kaplan–Meier method was used to determine the probability of survival in Graphpad Prism 5.0 for Windows.

## Results

### Differentially expressed genes (DEGs) and the heatmap

Based on the analysis of GSE1751 by R and the cut-off criteria, 37 differentially expressed genes were found between the pre-HD individuals and the healthy controls (Group 1) and 985 between the HD patients and the healthy controls (Group 2). There were 35 upregulated DEGs and 2 downregulated DEGs in group 1, and 756 upregulated DEGs and 229 downregulated DEGs in group 2. Additional file 1: Table S1 shows the gene expression level of all DEGs, indicating up- and downregulation as well as the expression differences between the two groups.

### Functional GO terms and pathway enrichment analysis

**Fig. 1:**
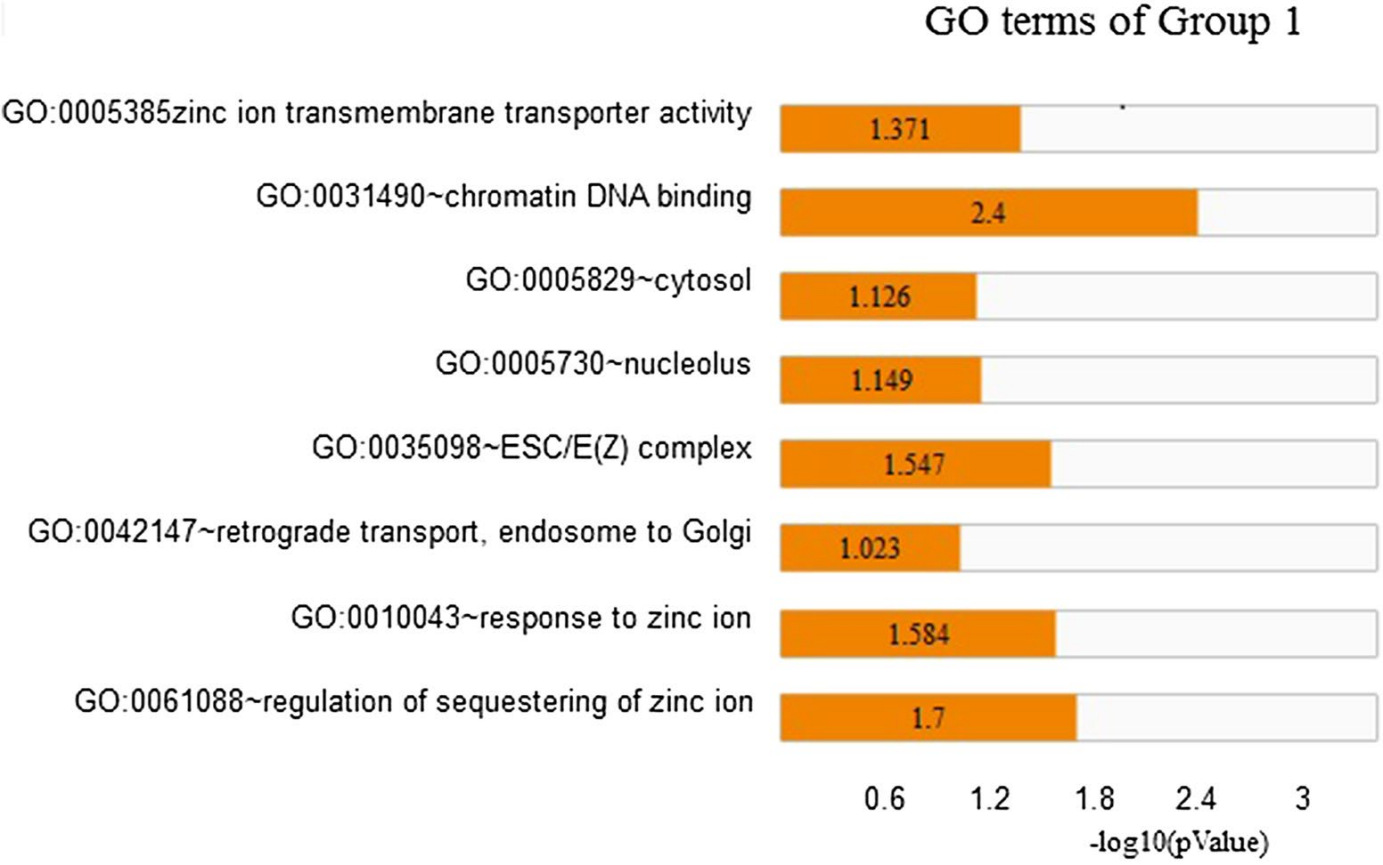
Gene ontology (GO) KEGG analysis of Group 1 (pre-HD & HC). The length of the orange bar represents the negative Log10 *p* value

**Fig. 2:**
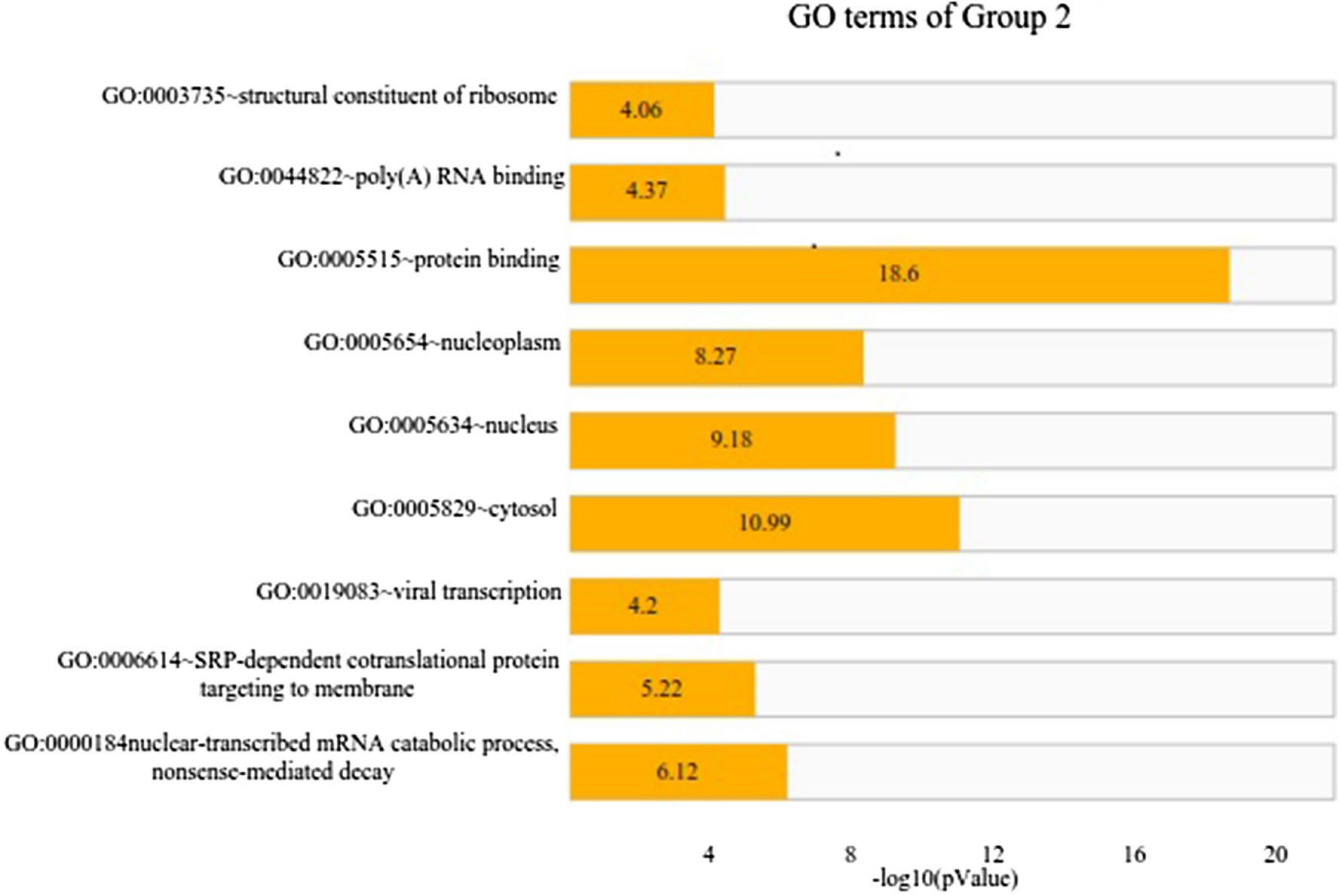
Gene ontology (GO) KEGG analysis of Group 2 (HD & HC). The length of the orange bar represents the negative Log10 *p* value

The top 3 GO terms related to the biological processes (BP) of the DEGs in Group 1 were regulation of sequestering of zinc ionic (fold enrichment: 94.78; *p*-value: 0.0201), response to zinc ion (Fold Enrichment: 72.91; *p*-value: 0.026), and retrograde transport, endosome to Golgi (Fold Enrichment: 19.34; *p*-value: 0.095). In Group 2, these BPs were nuclear-transcribed mRNA catabolic process, nonsense-mediated decay (Fold Enrichment: 3.56; *p*-value: 7.60E-07), SRP-dependent cotranslational protein targeting to membrane (Fold Enrichment: 3.68; *p*-value: 6.01E−06), and viral transcription (Fold Enrichment: 3.09; *p*-value: 6.38E−05). Moreover, the top 3 molecular function (MF) terms in Group 1 were chromatin DNA binding (Fold Enrichment: 30.31; *p*-value: 0.004) and zinc ion transmembrane transporter activity (Fold Enrichment: 44.26; *p*-value: 0.032). These MFs in Group 2 were protein binding (Fold Enrichment: 1.28; *p*-value: 2.49E−19), poly(A) RNA binding (fold enrichment: 1.54; *p*-value: 4.22E−5), and structural constituent of ribosome (Fold Enrichment: 2.34; *p*-value: 8.66E−5). These results show that zinc ion and chromatin DNA binding are involved in the pre-symptomatic stage and transcription and translation processes are important in the symptomatic stage (Figs. 1 and 2).

### Hub genes selected by PPI network analysis of DEGs

**Fig. 3:**
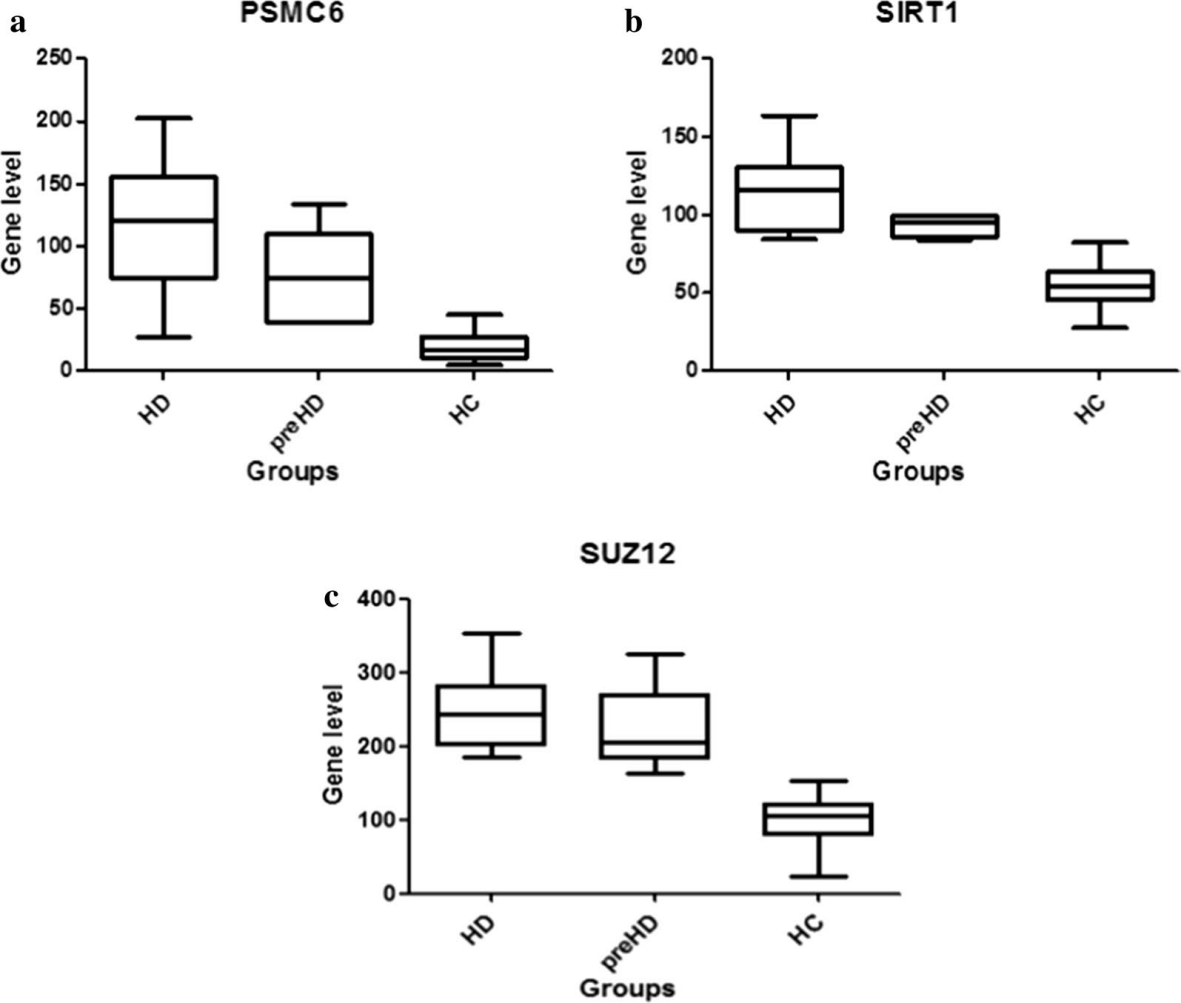
Box diagram showing the levels of the PSMC6 (**a**), SIRT1 (**b**), and SUZ12 (**c**) genes. The gene expression levels of PSMC6, SIRT1 and SUZ12 decreased on sequence HD group, preHD group and HC group

A total of 10 and 5394 nodes were identified from PPI network analysis of groups 1 and 2, respectively. Here, 3 hub genes were found in group 1 [sirtuin 1 (SIRT1), suppressor of zeste 12 (SUZ12), and proteasome 26S subunit, ATPase 6 (PSMC6)] and 14 were found in group 2 [fission mitochondrial 1 (FIS1), chromobox 1 (CBX1), zinc finger protein 217 (ZNF217), TATA-box binding protein-associated factor, RNA polymerase I subunit D (TAF1D) DEK, family with sequence similarity 60 member A(FAM60A), GABA type A receptor-associated protein like 2 (GABARAPL2), SIRT1, histone acetyltransferase 1 (HAT1), cyclin H (CCNH), histone deacetylase 2 (HDAC2), SUZ12, RRN3 homolog, RNA polymerase I transcription factor (RRN3), and PEST proteolytic signal-containing nuclear protein (PCNP)]. The genes and their scores are shown in Additional file 2: Table S2. Importantly, SIRT1 and SUZ12 are unregulated hub genes in both groups, but their expression levels were higher in group 2. The FC values of PSMC6 and SIRT1 in Group 2 were approximately twice those in Group 1 and 1.25 times that of the SUZ12 gene. The expression levels of these three hub genes in HD patients, pre-HD individuals, and healthy controls are shown in Fig. 3.

### GO and KEGG analysis results of hub genes in two groups

The functional enrichment results of the hub genes in the two groups are shown in Additional file 3: Table S3. Those in Group 1 mainly participate in the chromatin DNA binding process, and those in Group 2 play roles in the regulation of transcription, chromatin remodeling, cell cycle, proteasome-mediated ubiquitin-dependent protein catabolic processes, and histone-related metabolism processes.

**Fig. 4:**
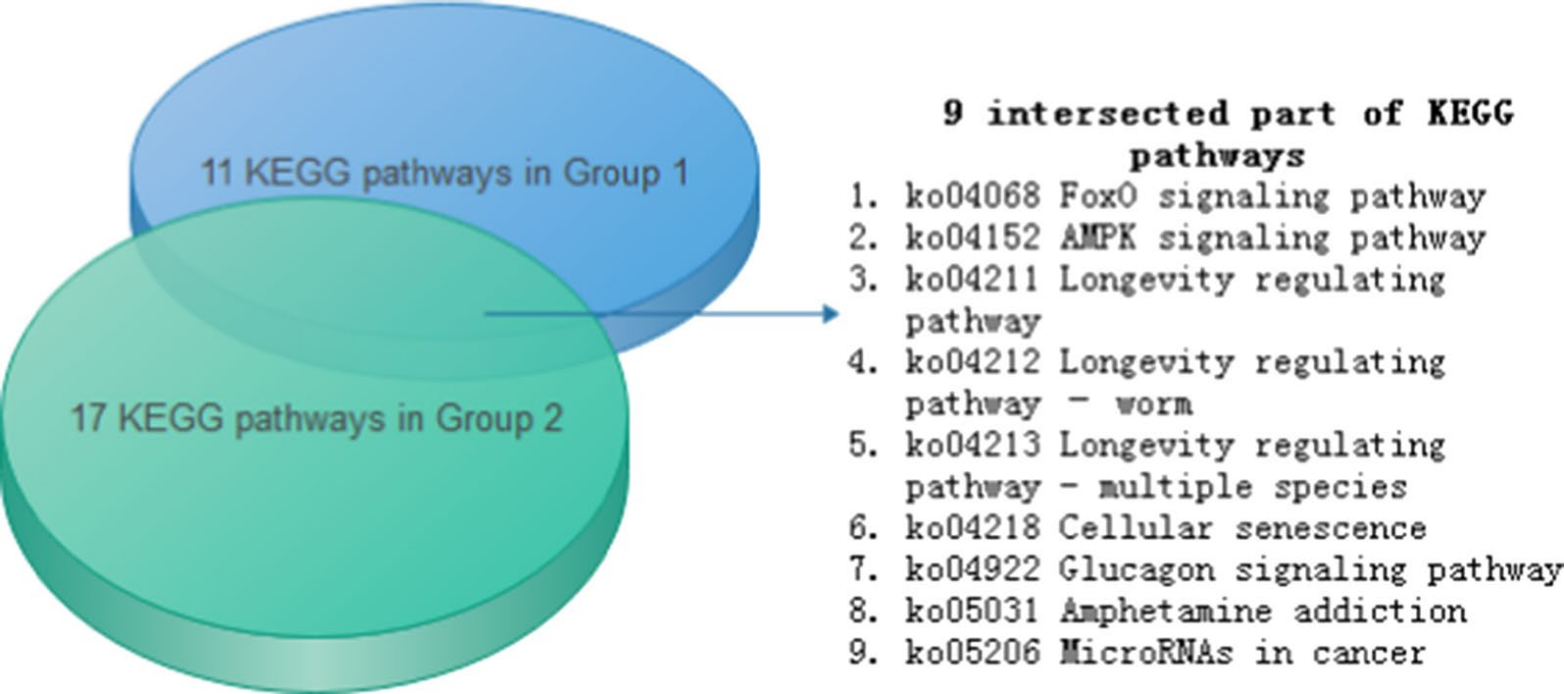
Venn diagram of the KEGG pathway results of the hub genes in the two groups. The blue cylinder represents group 1 and the green cylinder represents group 2. The intersection pathways are shown

The KEGG database was used for pathway analysis of the hub genes in the two groups. Those in Group 1 participate in 11 pathways, and those in Group 2 participate in 17 pathways. Among them, 9 pathways were intersected in the two groups (Fig. 4). All KEGG pathway results are shown in Additional file 4: Table S4.

### Prediction of miRNAs of the intersected hub genes and validation of hub genes

**Fig. 5:**
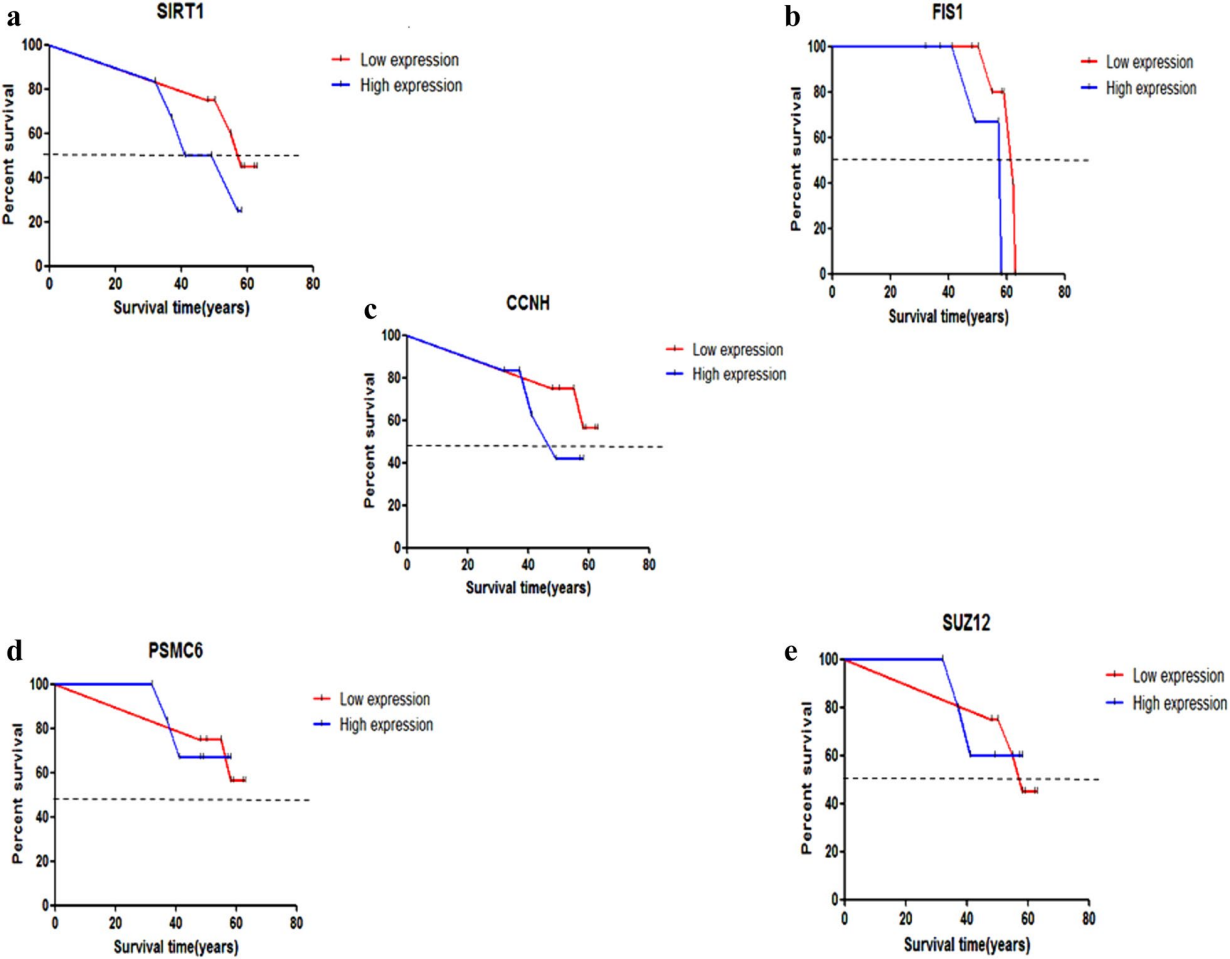
Kaplan–Meier analysis of the survival time for SIRT1 (**a**), FIS1 (**b**), CCNH (**c**), PSMC6 (**d**), and SUZ12 (**e**) in the validation dataset of 85 cases. The results show that the HD patients with high expression levels (blue line) of SIRT1, FIS1, and CCNH have a decreased overall survival time compared to those with low expression levels (red line).The remaining 2 genes showed no statistical significance between gene expression and clinical outcome of HD in the validation dataset. The dotted line represents the median survival time of those genes with different expression levels

Targetscan and miRDB were used to predict the miRNAs of the target genes, SIRT1 and SUZ12, and the intersected prediction results of these two databases became the final predicted miRNAs; 15 for SIRT1 and 13 for SUZ12. For the SIRT1 gene, 6 predicted miRNAs have previously been reported to have a clear relationship with HD (miR-22-3p, miR-138-5p, miR-9-5p, miR-132-3p, miR-135b-5p, and miR-135a-5p) [24, 25], and 1 miRNA for the SUZ12 gene (miR-19b-3p) [26]. All these results are shown in Table 1. To verify the 5 hub genes, the survival rate was calculated for the two groups in the validation dataset (GSE24250) through Kaplan–Meier analysis. We can see clearly that the HD patients with high expression levels of SIRT1, FIS1, and CCNH have a decreased overall survival time compared to those with low expression levels. Additionally, the Kaplan–Meier analysis curves of the high-expression group do not intersect with those of the low-expression group, indicating that the survival time of the high-expression group is always lower than that of the low-expression group. Figure 5a–c shows that the median survival time (dotted line) of the high-expression group is lower than that of the low-expression group. However, the P values of the three genes in the verification dataset are not statistically significant (0.2 for SIRT1, 0.1 for FIS1, and 0.2 for CCNH). This finding might be due to the small size of the validation dataset. The remaining 2 genes showed no statistical significance between gene expression and clinical outcome of HD in the validation dataset.

**Table 1:** The predicted and reported miRNAs of the SIRT1 and SUZ12 intersection genes

Predicted miRNAs of the SIRT1 gene	predicted miRNAs of the SUZ12 gene
hsa-miR-22-3p*	hsa-miR-3913-3p
hsa-miR-138-5p*	hsa-miR-19b-3p*
hsa-miR-9-5p*	hsa-miR-19a-3p
hsa-miR-6504-5p	hsa-miR-489-3p
hsa-miR-3064-5p	hsa-miR-520c-3p
hsa-miR-204-5p	hsa-miR-302b-3p
hsa-miR-211-5p	hsa-miR-302a-3p
hsa-miR-132-3p*	hsa-miR-520d-3p
hsa-miR-199b-5p	hsa-miR-520a-3p
hsa-miR-199a-5p	hsa-miR-302d-3p
hsa-miR-212-3p	hsa-miR-372-3p
hsa-miR-181d-5p	hsa-miR-302e
hsa-miR-30e-5p	hsa-miR-373-3p
hsa-miR-135b-5p*	
hsa-miR-135a-5p*	

The miRNAs marked with “*” have been reported previously

## Discussion

HD is a neurodegenerative disorder with a long symptomatic phase [27]. Although mHtt is expressed during embryonic development, clinical HD manifests in adulthood [28]. Various studies focusing on the early molecular pathogenesis in the pre-symptomatic stage may provide new insight into the therapeutic treatment of HD [29]; therefore, we attempted to elucidate hub genes, miRNAs, of pre-HD and HD. In recent years, bioinformatics has been increasingly applied to many fields including neurology, laying the foundation for further experimental verification [11, 30, 31]. In the present study, bioinformatics methods were used to predict hub genes and miRNAs associated with HD and pre-HD in both generation and validation datasets with the hope of providing insight into the pathogenesis in both HD and pre-HD patients. The results indicate that SIRT1, SUZ12, and PSMC6 may be involved in the pathogenesis of pre-HD, and FIS1, SIRT1, CCNH, and SUZ12 may play roles in symptomatic HD. GO term enrichment and KEGG analysis demonstrates that PSMC6 and its ubiquitin-mediated proteolysis pathway play an important role in pre-HD. Moreover, CCNH and transcriptional dysregulation, in addition to FIS1 and mitochondrial disruption, may participate in the pathogenesis of HD in the late stage. Interestingly, the intersected hub genes, SIRT1 and SUZ12, and their transcriptional regulation and histone-related metabolism pathways may play important roles in both pre-HD and HD. MiR-22-3p and miR-19b may be involved in the pathogenesis through these intersected hub genes, respectively.

Since no survival data regarding GSE1751 are available, an independent validated dataset, GSE24250, was employed to elucidate whether the up- or downregulation of hub genes could affect the survival time of HD patients. According to the Kaplan–Meier analysis results, the upregulation of SIRT1, FIS1, and CCNH has a negative correlation with survival time in HD patients, which is consistent with our results. However, the association of SUZ12 and PSMC6 with survival time was not statistically significant, which may be due to the low incidence of HD and the small sample size of our datasets.

In the present study, the PSMC6 gene was highly associated with the pre-symptomatic phase of HD. PSMC6, also called proteasome regulatory subunit 4 (RPT4), is a subunit of the 19S proteasome regulatory protein, which can participate in ubiquitin-mediated proteolysis [32, 33]. Disassembly of the proteasome by Htt aggregates has been reported in various studies [34], and overexpression of RPT4 has been shown to facilitate aggregation of mHtt in a cellular model of HD [35]. PSMC6 and the related ubiquitin-mediated proteolysis were shown to be involved in the pre-symptomatic stage. Interestingly, the expression level of PSMC6 in pre-HD patients was 74 as compared with 123 in HD patients and 20 in healthy controls, indicating that PSMC6 is already involved in the pre-symptomatic stage of HD and continues to play a role in the symptomatic stage.

Moreover, it was found that CCNH and FIS1 were highly associated with late-stage HD rather than the pre-symptomatic stage. CCNH is assembled with TFIIH core proteins and phosphorylates the C-terminal domain of RNA polymerase II to facilitate promoter clearance [36]. RNA polymerase II has been shown to increase in the postmortem HD brain [37] and in vitro [25]. We hypothesize that CCNH may play a role in the pathogenesis of HD. FIS1 could interact with dynamin-related protein 1 (DRP1), which is the primary component of mitochondrial fission [38]. An inhibitor of the DRP1–FIS1 interaction was protective in a mouse model of HD [39]. Therefore, CCNH and the related transcriptional dysregulation and FIS1 and the related mitochondrial disruption may be involved in the pathogenesis of HD during the symptomatic stage.

The SIRT1 and SUZ12 genes may play important roles during both the pre-symptomatic and symptomatic stage in HD patients. The FC values of SIRT1 and SUZ12 in Group 2 were approximately 2- and 1.25-times those in Group 1, respectively. SIRT1 belongs to a highly conserved family of sirtuins, the overexpression of which exerts neuroprotection through deacetylation of several transcription factors such as PCG-1α, p53, and FOXO3a [40–42]. The Sirt1 activator, resveratrol (RESV), decreases histone H3 acetylation at lysine 9 and improves motor coordination in the YAC128 and N171-82Q HD mouse model [43, 44]. SUZ12 is the core subunit of the polycomb repressive complex 2 (PRC2), which can implement gene silencing through methylation and ubiquitylation of histones [45–47]. There exists some evidence showing inhibition of PRC2 in HD through the upregulation of histone methylation with the participation of SUZ12 [48, 49]. Interestingly, another study also found that full-length huntingtin can stimulate the histone methylation of PRC2 [50]. Moreover, SUZ12 histone ubiquitylation and SIRT1-mediated deacetylation promote ubiquitin-dependent degradation [47, 51–53]. According to our functional enrichment results, SIRT1 and SUZ12 are involved in the pathogenesis of HD through several pathways, including transcriptional regulation, histone-related metabolism, and proteasome-mediated ubiquitin-dependent protein catabolic processes.

MiR-22-3p had the highest ranking among the predicted results for the SIRT1 gene, and has previously been reported to be directly related to HD in a mouse model [24]. Various studies have confirmed that the miR-22-3p/SIRT1 pathway plays an important role in the development of HD [42, 54]. As one of the predicted miRNAs of SUZ12, miR-19b has been reported previously. Although we failed to find an association between miR-19b and SUZ12, we still think that a correlation between miR-19b and SUZ12 may exist in HD [26]. More intriguingly, another 26 predicted miRNAs of SUZ12 and SIRT1 may also be related to the pathogenesis of HD, but further experiments are required.

## Conclusion

In our studies, PSMC6 and related ubiquitin-mediated proteolysis may participate in the pre-symptomatic phase of HD, while CCNH and related transcriptional dysregulation and FIS1 and related mitochondrial disruption may be involved in late-stage HD. SIRT1 and SUZ12 have been confirmed to play crucial roles from the pre-symptomatic to the symptomatic stage, and their associated transcriptional dysregulation, histone metabolism, and proteasome-mediated ubiquitin-dependent protein catabolic processes may be important. However, few studies have focused on the role of SUZ12 in HD to date, and further experiments are still required.

## Supplementary information

**Additional file 1: Table S1.** Differentially expressed genes involved in HD and preHD samples.

**Additional file 2: Table S2.** All of the hub genes and their scores in two groups.

**Additional file 3: Table S3.** Gene Ontology (GO) terms enrichment analysis of hub genes in Group 1 and Group 2.

**Additional file 4: Table S4.** KEGG pathways of the hub genes in two groups.

## Data Availability

GSE1751 and GSE24250 datasets were downloaded from GEO (https://www.ncbi.nlm.nih.gov/geo/)) [16, 55] and expression profiling arrays were generated using GPL96 (Affymetrix Human Genome U133 Plus 2.0 Array). R packages of “limma” (https://cran.r-project.org/), provided by a bioconductor project ( https://www.bioconductor.org/) [17], were applied to assess GSE1751 and GSE24250 RAW datasets. We applied online prediction tools utilizing Targetscan [21] (https://www.targetscan.org/vert_71/) and miRDB [22] (https://www.mirdb.org/), to predict potential microRNA targeting.

## Abbreviations

HD: Huntington’s disease
pre-HD: Pre-symptomatic HD
mHtt: Mutant huntingtin
DEGs: Differentially expressed genes
GEO: Gene expression omnibus
PPI: Protein–protein interaction
GO: Gene Ontology
KEGG: Kyoto Encyclopedia of Genes and Genomes
miRNA: MicroRNA
mRNA: Messenger RNA
RMA: Robust multi-array average
FDR: False discovery rate
FC: Fold changes
STRING: Search Tool for the Retrieval of Interacting Gene
MCODE: Molecular Complex Detection
DAVID: Database for Annotation, Visualization, and Integrated Discovery
BP: Biological processes
MF: Molecular function
SIRT1: Sirtuin 1
SUZ12: Suppressor of zeste 12
PSMC6: Proteasome 26S subunit, ATPase 6
FIS1: Fission mitochondrial 1
CCNH: Cyclin H
RPT4: Proteasome regulatory subunit 4
DRP1: Dynamin-related protein 1
RESV: Resveratrol
PRC2: Polycomb repressive complex 2
